# Emergence of KPC-113 and KPC-114 variants in ceftazidime-avibactam-resistant *Klebsiella pneumoniae* belonging to high-risk clones ST11 and ST16 in South America

**DOI:** 10.1128/spectrum.00374-23

**Published:** 2023-09-06

**Authors:** Felipe Vásquez-Ponce, Jessica Bispo, Johana Becerra, Herrison Fontana, Jesus G. M. Pariona, Fernanda Esposito, Bruna Fuga, Flavio A. Oliveira, Florencia Brunetti, Pablo Power, Gabriel Gutkind, Angelica Zaninelli Schreiber, Nilton Lincopan

**Affiliations:** 1 Department of Microbiology, Institute of Biomedical Sciences, University of São Paulo, São Paulo, Brazil; 2 One Health Brazilian Resistance Project (OneBR), São Paulo, Brazil; 3 Department of Clinical Analysis, School of Pharmacy, University of São Paulo, São Paulo, Brazil; 4 School of Medical Sciences, University of Campinas, Campinas, São Paulo, Brazil; 5 Facultad de Farmacia y Bioquímica, Instituto de Investigaciones en Bacteriologia y Virología Molecular, Universidad de Buenos Aires, and Consejo Nacional de Investigaciones Científicas y Técnicas (CONICET), Buenos Aires, Argentina; Laboratory Corporation of America Holdings, Burlington, North Carolina, USA

**Keywords:** antimicrobial resistance, KPC variants, ceftazidime-avibactam, *Enterobacterales*, international clones, genomic surveillance

## Abstract

Two novel variants of *Klebsiella pneumoniae* carbapenemase (KPC) associated with resistance to ceftazidime-avibactam (CZA) and designated as KPC-113 and KPC-114 by NCBI were identified in 2020, in clinical isolates of *Klebsiella pneumoniae* in Brazil. While *K. pneumoniae* of ST16 harbored the *bla*_KPC-113_ variant on an IncFII-IncFIB plasmid, *K. pneumoniae* of ST11 carried the *bla*_KPC-114_ variant on an IncN plasmid. Both isolates displayed resistance to broad-spectrum cephalosporins, β-lactam inhibitors, and ertapenem and doripenem, whereas *K. pneumoniae* producing KPC-114 showed susceptibility to imipenem and meropenem. Whole-genome sequencing and *in silico* analysis revealed that KPC-113 presented a Gly insertion between Ambler positions 264 and 265 (R264_A265insG), whereas KPC-114 displayed two amino acid insertions (Ser-Ser) between Ambler positions 181 and 182 (S181_P182insSS) in KPC-2, responsible for CZA resistance profiles. Our results confirm the emergence of novel KPC variants associated with resistance to CZA in international clones of *K. pneumoniae* circulating in South America.

**IMPORTANCE:**

KPC-2 carbapenemases are endemic in Latin America. In this regard, in 2018, ceftazidime-avibactam (CZA) was authorized for clinical use in Brazil due to its significant activity against KPC-2 producers. In recent years, reports of resistance to CZA have increased in this country, limiting its clinical application. In this study, we report the emergence of two novel KPC-2 variants, named KPC-113 and KPC-114, associated with CZA resistance in *Klebsiella pneumoniae* strains belonging to high-risk clones ST11 and ST16. Our finding suggests that novel mutations in KPC-2 are increasing in South America, which is a critical issue deserving active surveillance.

## OBSERVATION

*Klebsiella pneumoniae* carbapenemases (KPCs) have successfully spread in South America, becoming endemic in Argentina, Brazil, and Colombia, being associated with global clones belonging to sequence types (STs) ST11, ST25, ST258, ST307, ST340, ST437, ST512, and ST1271 (1 – 5). More recently, the international ST16 has also been identified in this region, being associated with an increased virulence potential (6 – 11).

For clinical treatment of infections caused for KPC-producing Enterobacterales, the combination of ceftazidime-avibactam (CZA) has been approved by the United States Food and Drug Administration (FDA) in 2015 (12). Worryingly, CZA resistance was reported early in 2015, in a KPC-3-producing *Klebsiella pneumoniae* of ST258 isolated in the United States of America, and it was related to the combination of OmpK36 mutation with increased KPC-3 expression (13, 14). Currently, CZA resistance has increased globally and is now associated with novel KPC variants (15 – 19). In this study, we report two novel KPC variants associated with resistance to CZA emerging in *K. pneumoniae* strains belonging to the high-risk clones ST11 and ST16, in Brazil, 2 years after CZA approval in this country (20).

In 2020, two CZA-resistant *K. pneumoniae* strains (330 and 331) were isolated from blood and rectal swab cultures from two different patients admitted to a teaching hospital.

Carbapenemase-positive *K. pneumoniae* strain 330 was isolated from a 61-year-old male patient admitted to the ICU ward with a medical history of alcoholic cirrhosis, hepatocellular carcinoma, hypertension, type 2 diabetes mellitus, and hepatic encephalopathy. The patient underwent a liver transplant. A surveillance rectal swab was collected, being negative for carbapenemase producers. However, after transplant, the patient’s respiratory status deteriorated, polymerase chain reaction COVID-19 test was negative, and based on chest X-ray abnormalities the patient was treated with polymyxin B, fluconazole, teicoplanin, meropenem, and sulfamethoxazole/trimethoprim. After a month of hospitalization, blood cultures tested positive for KPC-producing *K. pneumoniae* (strain 330), and pulmonary sepsis was the focus of the infection. The patient was treated with ceftazidime-avibactam, gentamicin, and sulfamethoxazole/trimethoprim. However, the patient’s condition deteriorated rapidly, with multiple organ dysfunctions, metabolic acidosis, and positive blood cultures for *Candida tropicalis*. Then therapy with ceftazidime-avibactam, polymyxin B, teicoplanin, and micafungin was started. However, clinical condition worsened and the patient died within 24 h.

Carbapenemase-positive *K. pneumoniae* strain 331 was isolated from a 59-year-old female patient with a medical history of chronic kidney disease, hypertension, smoking, and alcoholism. The patient was admitted to the nephrology ward for a kidney transplant. The patient did not experience any complications during hospitalization. A surveillance rectal swab was collected 1 week after transplant, and culture was negative for carbapenemase producers. However, a week later, a new surveillance rectal swab was collected being positive for carbapenemase-producing *K. pneumoniae* (strain 331). The patient was treated with meropenem for 14 days and a week later was discharged.

Both strains were identified by MALDI-TOF. Antimicrobial susceptibility testing was performed by BD Phoenix (Becton Dickinson), disk diffusion, broth microdilution, or MIC Test Strip (Liofilchem) methods, with interpretative criteria based on CLSI and/or EUCAST guidelines (21, 22). In this regard, both isolates displayed resistance to penicillins, cephalosporins, cephamycins, monobactam, and β-lactamase inhibitors (i.e., clavulanic acid, sulbactam, tazobactam, and avibactam), remaining susceptible to siderophore-linked cephalosporin. However, while strain 330 displayed resistance to imipenem and meropenem, strain 331 was susceptible to both carbapenems (Table 1).

**TABLE 1:** Antimicrobial resistance profile of *K. pneumoniae* strains producing KPC-113 and KPC-114 variants to β-lactam antibiotics

Antibiotics	Resistance profile (MIC, µg/mL)^*a*^	
	***K. pneumoniae*** 330 (KPC-113/ST16)	***K. pneumoniae*** 331 (KPC-114/ST11)
Ampicillin	R (>16)	R (>16)
Amoxicillin	R	R
Ticarcillin	R	R
Piperacillin	R	R
Amoxicillin/clavulanic acid	R (>16/8)	R (16/8)
Ampicillin/sulbactam	R	R
Ticarcillin/clavulanic acid	R (>64/2)	R
Piperacillin/tazobactam	R (>64/4)	I (16/4)
Ceftolozane/tazobactam	R (255/4)	R (12/4)
Ceftazidime/avibactam	R (32/4)	R (64/4)
Aztreonam	R (>16)	R (>16)
Cefoxitin	R	R
Cephalexin	R	R (>32)
Cephalothin	R	R
Cephazolin	R (>8)	R (>8)
Cefaclor	R	R
Cefuroxime	R	R
Cefixime	R	R
Cefoperazone	R	R
Cefotaxime	R	R
Cefpodoxime	R	R
Ceftazidime	R (>32)	R (>32)
Ceftriaxone	R (>32)	R (>32)
Cefepime	R (>16)	R (>16)
Ertapenem	R (>2)	R (1)
Doripenem	R	R
Imipenem	R (>8)	S (≤ 1)
Meropenem	R (>16)	S (≤ 0.25)
Cefiderocol	S (0.064)	S (0.25)
Amikacin	S (8)	S (4)
Ciprofloxacin	R (>64)	R (>64)

^*a*^ Resistance profile determined by disk-difussion method. Minimal inhibitory concentrations determined by Vitek system, broth microdilution, or MIC Test Strip method. Interpretative criteria were based on CLSI and EUCAST guidelines (21, 22).

The genomic DNA of *K. pneumoniae* strains was sequenced using the Illumina NextSeq platform (Illumina Inc., San Diego, CA), using the Nextera DNA Flex library prep and 2 × 75 bp paired-end reads. Quality-filtered reads were *De novo* assembled using Unicycler v0.4.8 (https://github.com/rrwick/Unicycler). Genomic analyses were performed using ABRicate v0.9.8 (https://github.com/tseemann/abricate) to predict antibiotic resistance genes (ResFinder 4.1), virulence genes profiling through the VFDB, and identification and typing of plasmid replicons (PlasmidFinder 2.1). Threshold ID and minimum length values (identity and coverage) of 90% were used for gene prediction.

**Fig 1:**
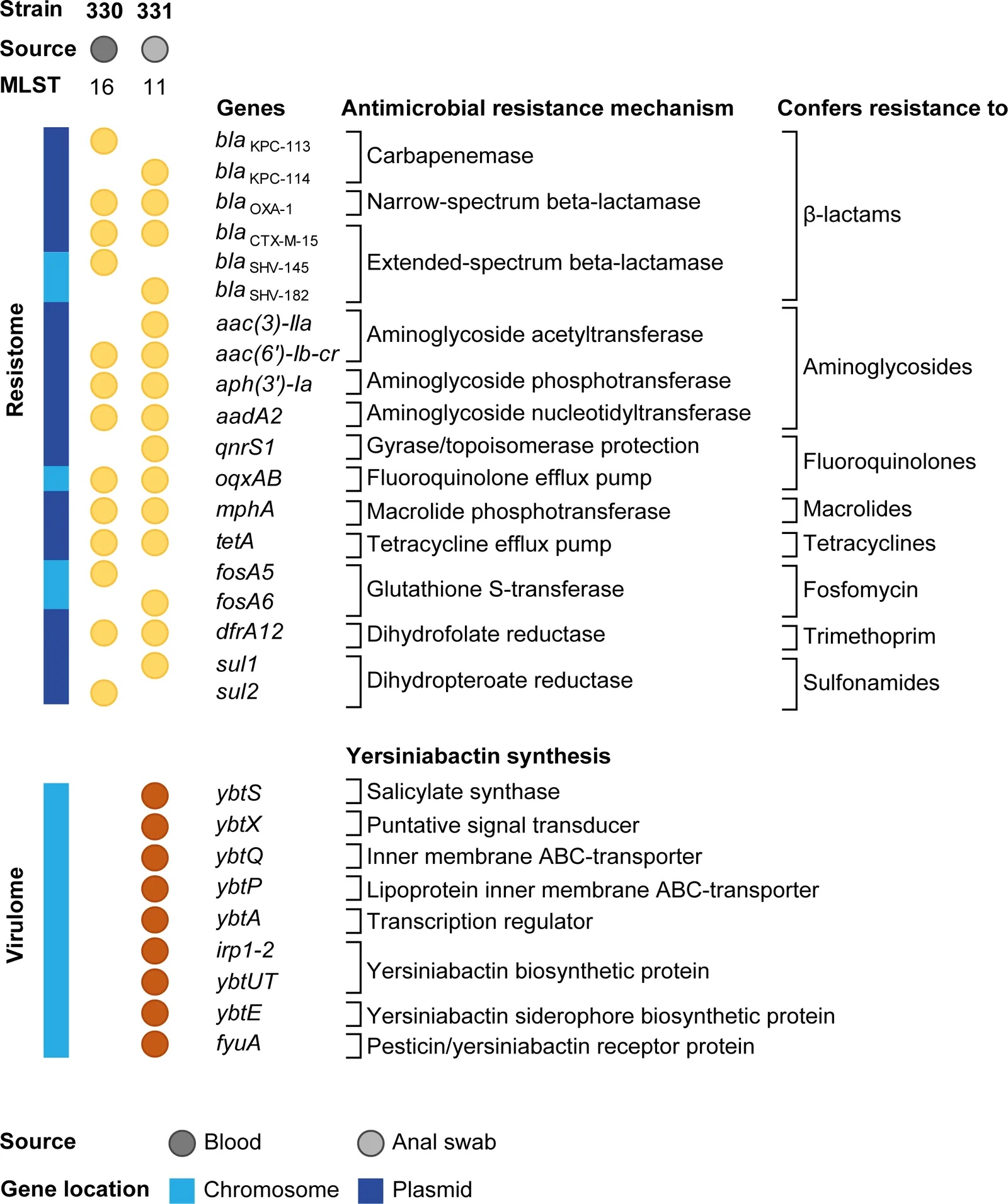
Heatmap displaying the resistome and virulome of *K. pneumoniae* 330/ST16 and *K. pneumoniae* 331/ST11 harboring KPC-113 and KPC-114, respectively. The colored regions represent the presence of antibiotic resistance genes (ARGs) and virulence genes (VGs) and the location (plasmid or chromosome). Blank fragments represent their absence.

Whole-genome sequencing analysis revealed that *K. pneumoniae* 330 (Bioproject ID: PRJNA867691; GenBank accession number: JANLGR000000000) belonged to ST16, and *K. pneumoniae* 331 (Bioproject ID: PRJNA868780; GenBank accession number: JANTNP000000000) belonged to ST11. Moreover, resistome analysis showed the presence of two novel KPC variants in *K. pneumoniae* 330 and 331, designated as KPC-113 (GenBank accession number: OM728506.1) and KPC-114 (GenBank accession number: OM728507.1), respectively, by NCBI. In addition, bioinformatic analysis showed that *bla*_KPC-113_ and *bla*_KPC-114_ are present in IncFII/IncFIB and IncN plasmids, respectively, associated with Tn*4401b*. The strain 330 carried chromosomal *bla*_SHV-145_, *oqxA*, *oqxB*, and *fosA5* genes and plasmidial *bla*_CTX-M-15_, *bla*_OXA-1_, *aph(3')-Ia, aac(6')-Ib-cr*, *aadA2*, *mphA*, *dfrA12*, *tetA,* and *sul2* genes. On the other hand, strain 331 carried *bla*_SHV-182_, *oqxA*, *oqxB,* and *fosA6* genes on the chromosome and *bla*_OXA-1_, *bla*_CTX-M-15_, *aac (3)-IIa*, *aph(3')-Ia*, *aac(6')-Ib-cr*, *aadA2*, *mphA*, *dfrA12*, *sul1*, *tetA,* and *qnrS1* on plasmids. Strikingly, only *K. pneumoniae* 331 belonging to ST11 harbored *ybtS, ybtX, ybtQ, ybtP, ybtA, irp2*, *irp1*, *ybtU*, *ybtT*, *ybtE*, and *fyuA* siderophore virulence genes (Fig. 1).

SNP-based phylogenomic analysis of *K. pneumoniae* strains 330 (ST16/KPC-113) and 331 (ST11/KPC-114), along with a collection of genomes from Brazilian *K. pneumoniae* strains, sharing identical STs (Table S1) was performed using CSI phylogeny v.1.4 (https://cge.food.dtu.dk/services/CSIPhylogeny/), revealing genomic relationship (83–176 SNP differences) of the KPC-113-producing *K. pneumoniae* with KPC-2-producing *K. pneumoniae* strains belonging to ST16, isolated from wound infection and blood cultures between 2014 and 2020. On the other hand, the KPC-114-producing *K. pneumoniae* displayed genomic relatedness (36–54 SNP differences) with two KPC-2-positive *K. pneumoniae* strains of ST11, isolated from blood cultures in 2015 and 2016 (Table S1).

A comparison of amino acid sequences with those of other KPC enzymes revealed that both KPC-113 and KPC-114 enzymes were novel allele variants of KPC-2. In this regard, KPC-113 presented a Gly insertion between Ambler positions 264 and 265 (R264_A265insG), whereas KPC-114 displayed two amino acid (Ser-Ser) insertions between Ambler positions 181 and 182 (S181_P182insSS) (Fig. S1).

In order to evaluate the *in vivo* activity of meropenem against meropenem-susceptible KPC-114-positive *K. pneumoniae* 331, healthy *Galleria mellonella* larvae weighing ~250 mg were selected and inoculated with 10 µL of 1.5 × 10^8^ CFU/mL K*. pneumoniae* 331. After 1 h, larvae were treated with 10 µL of 0.5 mg/mL meropenem or 10 µL of 1.0 mg/mL meropenem, in order to achieve clinical human doses of 1 g and 2 g meropenem, respectively, as standardized by the EUCAST for intravenous regimens (22, 23). The inoculum was delivered in the last right proleg, and the treatments were injected in the last left proleg by using a sterile insulin syringe. Five larvae were included in each tested group, and assays were performed in duplicate. While 60% of untreated *G. mellonella* larvae died at 48 h post-infection, 100% survival was observed in both *G. mellonella* groups treated with clinical doses of 1 g and 2 g meropenem (Fig. S2). Likewise, 100% survival was observed in the uninfected control group treated with 10 µL of sterile saline. Although this result suggests that meropenem could be an option for decolonization or treatment of meropenem-susceptible KPC-114-positive *K. pneumoniae*, additional investigation is necessary, in order to investigate the emergence of possible additional new mutations conferring resistance to CZA and meropenem. In this study, the colonized patient was treated with meropenem for 14 d and a week later was discharged, confirming favorable use of empiric therapy in KPC-positive *K. pneumoniae* colonized patients (24).

Carbapenem-resistant *K. pneumoniae* has been the most important species recovered from surveillance rectal swabs of hospitalized patients and the most common cause of subsequent infections (25, 26). In fact, colonization at multiple sites with carbapenem-resistant *K. pneumoniae* has been the strongest predictor of bloodstream infection development in previous large cohorts of carbapenem-resistant *K. pneumoniae* rectal carriers (27). In patients colonized by KPC-positive *K. pneumoniae*, utility of the Giannella Risk Score (https://www.pharmacyjoe.com/giannella-risk-score-calculator-for-infection-with-carbapenem-resistant-klebsiella-pneumoniae/) to predict infection risk has been previously confirmed (24, 28).

Following introduction of CZA into clinical use, emergence of bacterial resistance has been shortly reported (19). In this respect, among *K. pneumoniae* strains resistant to CZA, point mutations, insertions, and/or deletions have been described in various hot spots of *bla*_KPC-2_ and *bla*_KPC-3_ allele variants (19). Currently, more than 40 *bla*_KPC_ alleles conferring resistance to CZA have been reported, with most of the mutations being in the omega-loop (amino acid positions 164–179) (19). Specifically in South America, between 2021 and 2022 the number of novel KPC variants conferring resistance to CZA has rapidly increased, as reported in the NCBI database; with KPC-96, KPC-97, and KPC-115 (GenBank accession numbers: OK086970.1, OK086971.1, OM714909.1) being detected in Argentina, and KPC-103, KPC-104, KPC-105, KPC-106, KPC-107, KPC-108, KPC-139, KPC-140, KPC-141, KPC-142, and KPC-143 (GenBank accession numbers: OL445423.1, OL445424.1, OL445426.1, OL445428.1, OL445425.1, OL445427.1, OP503887.1, OP503888.1, OP503889.1, OP503890.1, OP503891.1) being now identified in Brazil.

In this study, we described the emergence of KPC-113 e KPC-114 variants associated with CZA resistance, originating from ST11 and ST16, the most threatening and widespread KPC-2-producing clones in South America (5 – 10). Noteworthy, R264_A265insG in KPC-113 was outside the omega-loop region of the KPC-2 protein, whereas S181_P182insSS in KPC-114 was near the omega-loop region of the KPC-2. In this regard, amino acid substitutions outside the omega-loop region of KPC-2 have also been associated with CZA resistance, such as in KPC–8,–14, –23,–28, –29,–41, –44,–50, –58,–63, –67,–74, –79,–80, –82,–84, –87,–96, –97,–103, and −105 variants (19).

Strikingly, KPC-114 was not detected by the NG-Test CARBA 5 lateral flow immunochromatographic test (NG Biotech) (29). Previous studies have demonstrated that KPC-31, KPC-33, KPC-68, KPC-71, KPC-78, KPC-90, KPC-104, KPC-106, KPC-139, KPC-141, KPC142, and KPC-143 variants also have been not detected by NG-Test CARBA-5 (30, 31). Therefore, most likely R264_A265insG and S181_P182insSS mutations confer protein conformations leading to inefficient binding of immobilized monoclonal antibodies targeted to recognize KPC-type enzymes inhibited by avibactam, consequently resulting in the failure of the detection method. On the other hand, CZA-resistant isolates displaying susceptibility to meropenem could not be identified as KPC producers (32), leading to a misleading detection by diagnostic laboratories. Thus, since CZA has become the first-line option for the treatment of infections due to KPC-2 producers, it is imperative to improve screening methods for the detection of KPC variants displaying resistance to CZA, in order to facilitate its rapid and accurate detection.

Although a limitation of this study is the lack of kinetic data, shortly after NCBI designation of the novel *bla*_KPC_ allele identified in the CZA-resistant *K. pneumoniae* strain 330 as *bla*_KPC-113_ (GenBank accession number: OM728506.1), a report from China described the presence and kinetic properties of KPC-113, identified in *Pseudomonas aeruginosa*, confirming considerable hydrolyzing abilities to carbapenems and ceftazidime and the significantly weakened inhibitory effect of avibactam (33).

In summary, we described two novel KPC variants, KPC-113 and KPC-114, associated with resistance to CZA in high-risk clones of *K. pneumoniae* belonging to ST11 and ST16. Our finding suggests that novel mutations in KPC-2 are increasing in South America, which is a critical issue deserving active surveillance.

## Data Availability

The genome sequences of *K. pneumoniae* 330 (ST16) and *K. pneumoniae* 331 (ST11) were deposited at GenBank under the accession numbers JANLGR000000000 and JANTNP000000000, respectively.

## AFTER EPUB

*[This article was published on 6 September 2023 with errors in reference 30. The reference was corrected in the current version, posted on 26 September 2023.]*

## SUPPLEMENTAL MATERIAL

Fig S1 spectrum.00374-23-s0001.docx Amino acid sequence alignment of KPC-2, KPC-113, and KPC-114 variants.

Fig S2 spectrum.00374-23-s0002.docx Survival curves of *Galleria mellonella* larvae infected with KPC-114-positive *K. pneumoniae* 331 and treated with meropenem.

TABLE S1 spectrum.00374-23-s0003.xlsx Accession numbers, epidemiological data and resistome of *K. pneumoniae* strains belonging to ST11 and ST16 circulating in Brazil from 2006.

ASM does not own the copyrights to Supplemental Material that may be linked to, or accessed through, an article. The authors have granted ASM a non-exclusive, world-wide license to publish the Supplemental Material files. Please contact the corresponding author directly for reuse.
