# Supplementary material for: Emergence of KPC-113 and KPC-114 variants in ceftazidime-avibactam-resistant Klebsiella pneumoniae belonging to high-risk clones ST11 and ST16 in South America
Source: Microbiol Spectr. 2023 Sep 6;11(5):e00374-23. doi: 10.1128/spectrum.00374-23 (PMC10580961; doi:10.1128/spectrum.00374-23)
Supplement: Fig S1 — Amino acid sequence alignment of KPC-2, KPC-113, and KPC-114 variants. [file spectrum.00374-23-s0001.docx]

**Figure S1**

**Figure S1.** Amino acid sequence alignment of KPC-2, KPC-113 and KPC-114 variants. KPC-113 presents a Gly insertion between Ambler position 264 and 265 (R264_A265insG), whereas KPC-114 displays two amino acid (Ser-Ser) insertions between Ambler position 181 and 182 (S181_P182insSS), associated with CZA resistance in *K. pneumoniae* 330 (ST16) and 331 (ST11) strains.
