# Supplementary material for: Emergence of KPC-113 and KPC-114 variants in ceftazidime-avibactam-resistant Klebsiella pneumoniae belonging to high-risk clones ST11 and ST16 in South America
Source: Microbiol Spectr. 2023 Sep 6;11(5):e00374-23. doi: 10.1128/spectrum.00374-23 (PMC10580961; doi:10.1128/spectrum.00374-23)
Supplement: Fig S2 — Survival curves of Galleria mellonella larvae infected with KPC-114-positive K. pneumoniae 331 and treated with meropenem. [file spectrum.00374-23-s0002.docx]

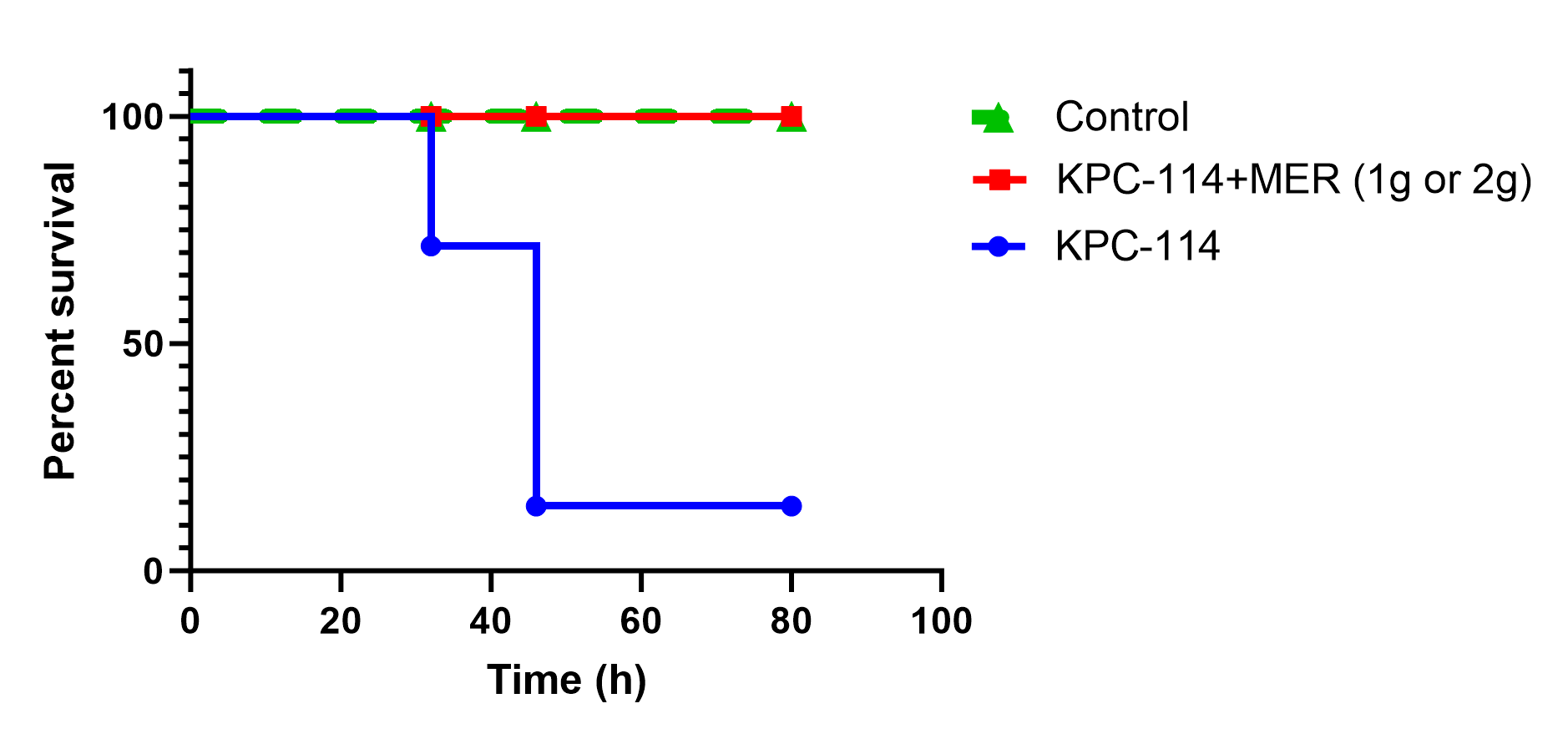
**Figure S2**

**Figure S2.** Survival curves of *Galleria mellonella* larvae infected with 10 μL of 1.5 × 10^8^ CFU/mL of meropenem susceptible KPC-114 positive *K. pneumoniae* 331 (blue line) and treated with 1g or 2g of meropenem (red line) after 1 hour post inoculation of KPC-114 positive *K. pneumoniae* 331. Uninfected control group was treated with 10 µL of sterile saline (green dashed line). 60% untreated *G. mellonella* larvae died at 48h post infection and 100% of survival was observed in both *G. mellonella* groups treated with clinical doses of 1g and 2g meropenem.
